# Hemagglutinin glycosylation pattern-specific effects: implications for the fitness of H9.4.2.5-branched H9N2 avian influenza viruses

**DOI:** 10.1080/22221751.2024.2364736

**Published:** 2024-06-07

**Authors:** Yixue Sun, Yanting Zhu, Pengju Zhang, Shouzhi Sheng, Zhenhong Guan, Yanlong Cong

**Affiliations:** a Department of Policies and Regulations, Changchun University, Changchun, People’s Republic of China; b State Key Laboratory for Diagnosis and Treatment of Severe Zoonotic Infectious Diseases, Key Laboratory for Zoonosis Research of the Ministry of Education, and College of Veterinary Medicine, Jilin University, Changchun, China; c Institute of Animal Biotechnology, Jilin Academy of Agricultural Sciences, Changchun, People’s Republic of China

**Keywords:** Avian influenza, H9N2 subtype, h9.4.2.5 branch, hemagglutinin, glycosylation, adaptive phenotype

## Abstract

Since 2007, h9.4.2.5 has emerged as the most predominant branch of H9N2 avian influenza viruses (AIVs) that affects the majority of the global poultry population. The spread of this viral branch in vaccinated chicken flocks has not been considerably curbed despite numerous efforts. The evolutionary fitness of h9.4.2.5-branched AIVs must consequently be taken into consideration. The glycosylation modifications of hemagglutinin (HA) play a pivotal role in regulating the balance between receptor affinity and immune evasion for influenza viruses. Sequence alignment showed that five major HA glycosylation patterns have evolved over time in h9.4.2.5-branched AIVs. Here, we compared the adaptive phenotypes of five virus mutants with different HA glycosylation patterns. According to the results, the mutant with 6 N-linked glycans displayed the best acid and thermal stability and a better capacity for multiplication, although having a relatively lower receptor affinity than 7 glycans. The antigenic profile between the five mutants revealed a distinct antigenic distance, indicating that variations in glycosylation level have an impact on antigenic drift. These findings suggest that changes in the number of glycans on HA can not only modulate the receptor affinity and antigenicity of H9N2 AIVs, but also affect their stability and multiplication. These adaptive phenotypes may underlie the biological basis for the dominant strain switchover of h9.4.2.5-branched AIVs. Overall, our study provides a systematic insight into how changes in HA glycosylation patterns regulate the evolutionary fitness and epidemiological dominance drift of h9.4.2.5-branched H9N2 AIVs, which will be of great benefit for the glycosylation-dependent vaccine design.

## Introduction

Influenza A virus (IAV) is an enveloped virus belonging to the genus *Alphainfluenza*, family *Orthomyxoviridae*, with eight single negative-strand RNA genome, which poses a serious threat to both avian and mammalian species [1]. During the long-term battle between IAV and the host, the host progressively strengthens its defenses against IAV infection by activating innate or acquired immunity, while IAV has evolved multiple strategies to circumvent the host's multiple lines of immune defense [2]. Hemagglutinin (HA), an IAV surface glycoprotein, initiates viral entry by binding to the sialic acid (SA) receptors on the cell surface and serves as the primary target for IAV vaccines [3, 4]. Compared to other IAV proteins, HA evolves at the highest rate [5]. The ability of N-linked glycosylation, a post-translational modification, to attach host-derived glycans to the globular head and stem of the HA ectodomain, sterically shields or exposes the functional regions of HA. This, in turn, mediates the evolution of IAV and the production of viral variants [6–8].

The degree of HA glycosylation of H1N1 and H3N2 IAVs has been demonstrated to tend to increase with time, particularly on the HA head [9]. It is essential for viral adaptive evolution [6, 10, 11]. However, HA does not undergo glycosylation modification without restraint, as it has to take the resulting fitness costs into account. Once a certain level of glycosylation is reached, HA would reposition its glycans or change the degree of glycosylation to constantly regulate the functional balance between receptor affinity and immune evasion so as to maintain viral fitness [10, 12, 13]. Understanding how HA glycosylation patterns affect viral adaptive phenotypes will therefore provide light on the dynamics of IAV adaptive evolution and lay the foundation for the development of the next-generation vaccines that take advantage of glycosylation-dependent mechanisms.

Since the 1990s, H9N2 avian influenza viruses (AIVs) have been enzootic in poultry across Asia and North Africa [14]. Due to their highly contagious and insidious nature, H9N2 AIVs continue to threaten the poultry industry, causing inestimable economic losses [15–18]. Noteworthily, this subtype of virus has broken through the interspecies barrier to transmit to humans [19]. Since 2007, H9N2 AIVs have become more and more homogeneous, with the emergence of h9.4.2.5-branched AIVs with absolute dominance [20, 21]. The molecular details underlying the fitness of this branch of viruses, meanwhile, have not yet been thoroughly elucidated. Given the critical role of glycosylation in HA functions, we investigated the effects of the genetic polymorphisms of HA glycosylation patterns on the adaptive phenotypes of h9.4.2.5-branched AIVs.

## Materials and methods

### Cells

Primary chicken embryo fibroblast (CEF) cells were prepared from 9-day-old specific-pathogen-free (SPF) embryonated chicken embryos as previously described [22]. CEF, Madin-Darby canine kidney (MDCK) cells (ATCC, CCL-34) and human embryonic kidney (HEK 293 T) cells (ATCC, CRL-11268) were cultured in Dulbecco’s modiﬁed Eagle medium (Gibco, USA) supplemented with 10% fetal bovine serum (Gibco, USA) and antibiotics at 37°C with 5% CO_2_.

### Phylogenetic analysis

The HA gene sequences of H9N2 AIVs isolated from China (n = 6,274) were downloaded from the GenBank (https://www.ncbi.nlm.nih.gov/genomes/FLU/Database/nph-select. cgi?go = database) and GISAID (http://platform.gisaid.org) databases (data as of December 31, 2022). A phylogenetic tree of the HA gene was constructed based on the maximum-likelihood method in MEGA software version 11.0 [23].

### Construction of expression plasmids and of generation reverse genetic viruses

Based on an h9.4.2.5-branched H9N2 virus strain (GISAID No. EPI ISL 374042), five HA gene mutants with different glycosylation patterns were constructed by the site-directed mutagenesis and cloned into the expression plasmid pCAGGS. The five mutant viruses were rescued using reverse genetic techniques [24]. Briefly, the HA gene and the remaining 7 genes were cloned into pHW2000, respectively. The eight plasmids were transfected into 293 T cells using Lipofectamine 3000 (Invitrogen, USA). The cells were incubated at 37°C in 5% CO_2_ and supplemented with 2 μg/mL of TPCK-treated trypsin (Sigma, USA) after 24 h. The supernatant of cells was collected and inoculated into 9–11-day-old SPF embryonated chicken eggs. After inoculation for 72 h, the allantoic fluid was collected to determine viral hemagglutination titres. The HA genes of the five virus mutants were then identified by sequencing.

### Deglycosylation of HA

To determine the occupancy by glycans in the predicted N-linked glycosylation sites (NGSs) on HA, the HA protein with a specific HA glycosylation pattern was co-incubated with PNGase F enzyme (New England Biolabs, USA) according to the manufacturer's instructions to remove the glycans from the HA protein under the denaturing conditions. Briefly, pCAGGS-HA was transfected into 293 T cells using Lipofectamine 3000 (Invitrogen, USA), and the cells were collected after 48 h. In the cell product, 10× Glycoprotein Denaturing Buffer (New England Biolabs, USA) was added. After 10 min of boiling at 100°C, the mixture was centrifuged for 10 s at 12,000 rpm. The resultant supernatant was then supplemented with NP40 (New England Biolabs, USA), GlycoBuffer 2 (New England Biolabs, USA), followed by the addition of PNGase F enzyme. After 1 h of incubation at 37°C, the mixture was subjected to a Western blot analysis, probed with HA-Tag monoclonal mouse antibody (Abmart, China) against the HA protein and goat anti-mouse antibodies (Abmart, China). The band shifts were used to validate the presence or absence of glycosylation at differential NGSs between the five HA mutants.

### Erythrocyte elution assay

The erythrocyte elution assay was performed as previously described [25]. Briefly, a twofold dilution of 128 HAU of each virus was mixed with an equal volume of 0.5% RBCs in a 96-well hemagglutination plate. After incubation at 4°C for 1 h, the hemagglutination titres were recorded every 30 min at 33°C for 6 h. The dissociation curves were plotted with the incubation time at 33°C as the horizontal coordinate and the hemagglutination titre as the vertical coordinate to determine the viral ability to bind to RBCs.

### Receptor affinity assay

The receptor affinity assay was performed according to the protocol described by Peacock et al. [14]. Brieﬂy, 1% chicken RBCs were treated at 37°C for 1 h with twofold serial dilutions of the RDE (Vibrio cholerae neuraminidase) (Sigma, USA) to remove SAs. RBCs were then washed three times and prepared into a 1% suspension. 50 μL of RDE-treated RBCs were incubated with an equal volume of 4 HAU of virus on ice for 45 min to record the highest concentration of RDE that can allow complete hemagglutination.

### Solid-phase binding assay

The solid-phase binding assay using a streptavidin–biotin detection system was performed as previously described [26]. Briefly, a 96-well ELISA plate was incubated overnight at 4°C with 128 HAU/50 μL of each virus. After washing and blocking, the plate was incubated with 2-fold serial dilutions of Neu5Acα2,3Galβ1,4GlcNAc-SpNH-PAA-biotin (3′SLN) or Neu4Acα2,6Galβ1, 4GlcNAc-SpNH-PAA-biotin (6′SLN) (Consortium for Functional Glycomics, USA) at 4°C for 1 h. After incubation with horseradish peroxidase-conjugated streptavidin (Roche, Germany) for 1 h, a positive reaction was visualized by the addition of a substrate solution containing TMB. The absorbance value at 450 nm was determined on a microplate reader (Labsystems, Finland).

### Generation of antisera

Antisera were prepared as previously described [14]. The 3-week-old SPF white Leghorn chickens (Beijing Merial Vital Laboratory Animal Technologies Co., LTD, China) were immunized with 0.5 mL of 0.1% paraformaldehyde-inactivated virus in the presence of Montanide adjuvant. Three chickens were immunized with each virus. On day 21 after immunization, the collected sera were inactivated at 56°C for 30 min and pretreated with RDE overnight at 37°C to remove nonspecific inhibitors. The pooled sera were stored at – 20°C until use.

### Cross-HI assay

The cross-HI assay was performed as previously described [27]. Briefly, 8 HAU/50 μL of each virus was added to serially twofold diluted sera. After incubation at 37°C for 1 h, 50 μL of 1% RBCs were added. The homologous and heterologous HI titres were expressed as the reciprocal of the highest serum dilution that completely inhibited hemagglutination and converted into log_2_ values.

### Construction of a viral antigenic map

A viral antigenic map was constructed by referring to the method as described by Smith et al. [28]. Briefly, the cross-HI dataset consisted of 15 antisera against the five viruses. The result was expressed as a 15 × 5 matrix, with each row representing the difference in HI titres between each of the five viruses against each of the 15 antisera. The 15 × 5 matrix was normalized to have a mean of 0 and an SD of 1 for each virus against each of the 15 antisera. Accordingly, we calculated the distances between the five mutants using the following formula and obtained the resulting symmetric 5 × 5 distance matrix. From this 5 × 5 distance matrix, the distances between any three viruses were compared by extracting the 3 × 3 distance submatrix and solving the distance equation to obtain the x, y coordinates of each virus for the antigenic map.

$$\mathrm{dist}=\sqrt{\overset{15}{\underset{k=1}{\sum }}{\left(m_{i,j}-m_{i,j}\right)}^{2}}$$

### pH stability

H9N2 virus with 128 HAU/50 µL was mixed with an equal volume of 100 mM acetate buffer (pH 4.0 and 5.0), 100 mM phosphate buffer (pH 6.0), or 100 mM neutral phosphate buffer (pH 7.0) as previously described [29]. After incubation at 37°C for 10 min, viral titres were determined by hemagglutination assay [30].

### Thermostability assay

The thermostability assay was performed as described [31]. Briefly, 128 HAU/50 µL of virus was incubated at 33°C or 37°C for 2 , 4 , 8 , 12 , 24 , 48 and 72 h. The heat-treated virus samples were then cooled to 4°C, and a hemagglutination assay was performed in triplicate to determine the change in viral hemagglutination titres.

### Viral growth kinetics

The TCID_50_ was determined by diluting each virus in a tenfold gradient with five replicate wells as described by the Reed-Muench method [32] and converted to the corresponding MOI. Briefly, CEF cells in 96-well plates were infected with each virus at an MOI of 0.1 or 0.01. Cell supernatants were collected every 2 h for 12 h or every 12 h for 72 h after infection to determine the viral TCID_50_ at different time points.

### Statistical analysis

The data were analyzed using GraphPad Prism 8.0 software. *, **, ***, and **** indicate *p *< 0.05, *p *< 0.01, *p *< 0.001, and *p *< 0.0001, respectively.

## Results

### The epidemiological dominance of H9N2 AIVs

**Figure 1.**
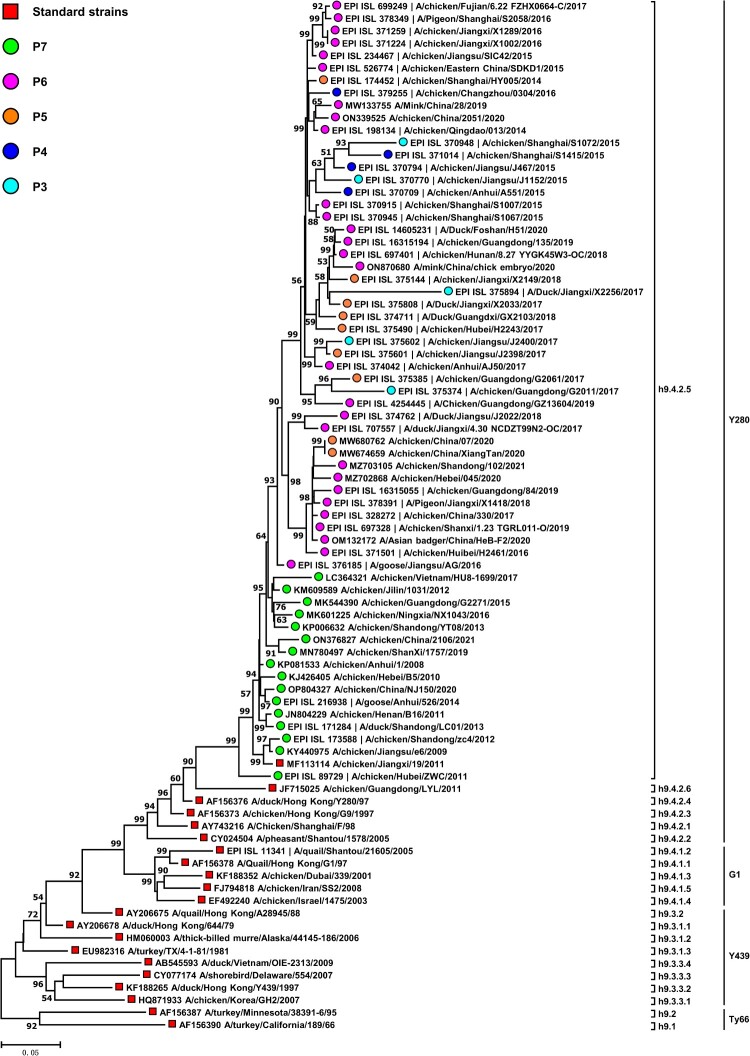
A simplified version of the phylogenetic tree of the HA genes of H9N2 viruses. The original phylogenetic tree based on the full-length HA sequences of 6,274 H9N2 AIV strains isolated from China was constructed by MEGA version 11 (http://www.megasoftware.net/) using a maximum-likelihood method with the Kimura 2-parameter model and 1,000 bootstrap replicates. For visualization, we randomly selected part of 6,274 H9N2 AIV strains to show a simplified version. The red squares represent standard strains in each branch of H9N2 viruses, whereas the coloured circles indicate representative strains of H9N2 viruses with different HA glycosylation patterns in the h9.4.2.5 branch. Bootstrap values >50% are shown at the branch nodes. The red squares on the tree represent the standard strains. The branches and sublineages are based on the nomenclature of Jiang et al. [60] and Deng et al. [61].

The number and circulating duration of viral strains within various branches of the HA phylogenetic tree were enumerated, which was constructed using 6,274 H9N2 AIV isolates from China. To present a clear HA phylogeny, we selected some strains as representative from each branch to construct a simple phylogenetic tree. According to Figure 1 and Table 1, the virus strains from the North American lineage, represented by the Ty66 sublineage (consisting of h9.1 and h9.2 branches), were uncommon in poultry flocks as compared to those strains from the Eurasian lineage, represented by the sublineages of Y439 (h9.3.1.1 to h9.3.3.4 branches), G1 (h9.4.1.1 to h9.4.1.5 branches), and Y280 (h9.4.2.1 to h9.4.2.6 branches). It is noteworthy that the h9.4.2.5-branched viruses within the Y280 sublineage accounted for 85.72% (n = 5,378) of the total number (n = 6,274) of H9N2 virus strains isolated in China since 1976, indicating the absolute dominance of this branch.

**Table 1.** Analysis of the dominant branch of H9N2 viruses isolated in China during 1976–2022.

Lineages	Sublineages	Branches	No. of strains	Years of isolation
American lineage	Ty66	h9.1 and h9.2	3	2000, 2008, 2012
Eurasian lineage	Y439	h9.3.1.1 to h9.3.3.4	47	1976–2016
	G1	h9.4.1.1 to h9.4.1.5	43	1997–2017
	Y280	h9.4.2.1 to h9.4.2.4, h9.4.2.6	803	1994–2017
		h9.4.2.5	5,378	2007–2022

### The dominance of h9.4.2.5-branched H9N2 AIVs with different HA glycosylation patterns

**Figure 2.**
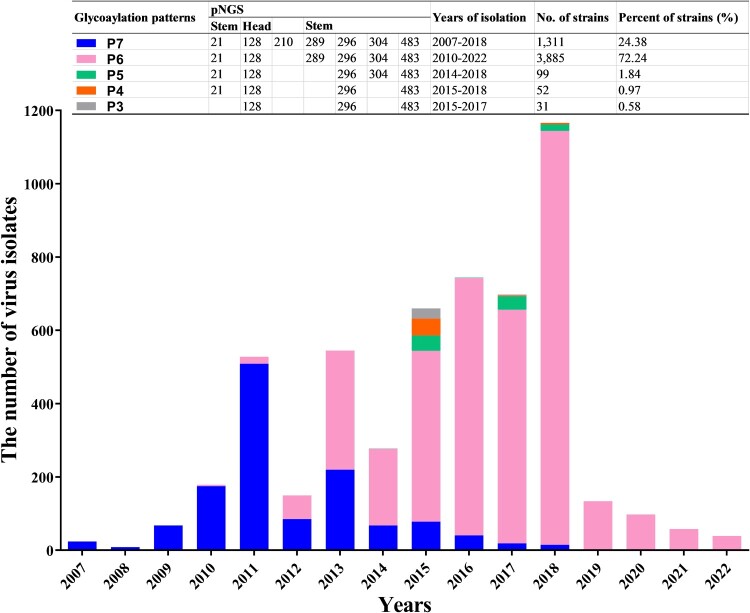
Statistics on the dominant HA glycosylation patterns of h9.4.2.5-branched H9N2 AIV strain during 2007–2022. The coloured rectangles represent different HA glycosylation patterns.

Up to seven NGSs (Asn-X-Ser/Thr-Y, where X/Y can be any amino acid except proline) [33] on the HA of 5,378 strains of h9.4.2.5-branched AIVs were found at positions 21, 128, 210, 289, 296, 304, and 483 (H3 numbering used throughout). The NGSs at positions 21, 289, and 304 were relatively conserved, whereas those at positions 128, 296 and 483 were absolutely conserved (Figure S1). These seven NGSs formed five major glycosylation patterns, termed P3 to P7. As shown in Figure 2, viruses with seven NGSs were first discovered in 2007, followed by a series of viruses with six to three NGSs after 2010. It is notable that viruses with different glycosylation patterns showed distinct epidemiological dominance. For instance, up until 2012, viruses with seven NGSs dominated the strain population; however, in 2013, viruses with six NGSs at positions 21, 128, 289, 296, 304, and 483 quickly surpassed them in number. Thereafter, the glycosylation pattern with six NGSs has become the dominant glycosylation phenotype of h9.4.2.5-branched AIVs. In contrast, only a few virus strains with five to three NGSs were isolated each year.

### Identification of the HA glycosylation of h9.4.2.5-branched H9N2 AIVs

**Figure 3.**
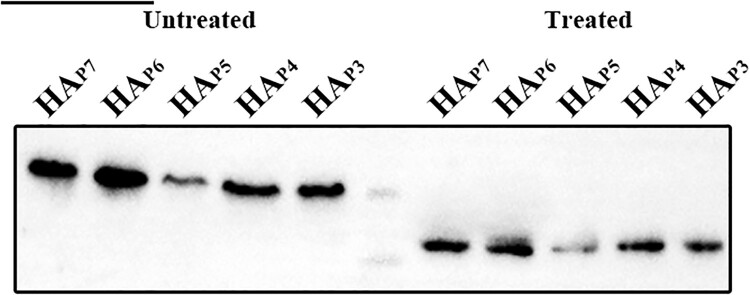
Glycosylation of non-conserved glycosylation sites at positions 210, 289, 304, and 21 identified by Western blotting. The five HA proteins with different glycosylation patterns that were treated without and with PNGase F are shown in lanes 1–5 and lanes 7–11. The marker in Lane 6 is 75 and 65 kDa from top to bottom.

Among the five HA glycosylation patterns, the corresponding NGS was absent at positions 210, 289, 304, and 21 of P6 to P3, respectively (Table S1). Nevertheless, each NGS in each glycosylation pattern had the potential to be glycosylated using the NetNGlyc web tool, available at https://services.healthtech.dtu.dk/services/NetNGlyc-1.0/. As glycosylation modifications only occur on the globular head and stem of the HA extracellular domain [34], five HA plasmid mutants with different HA glycosylation patterns were constructed and named HA_P7_, HA_P6_, HA_P5_, HA_P4_, and HA_P3_. To verify that the NGSs of each glycosylation pattern were glycosylated, we then compared the protein migration rates of these five HA mutants treated with or without N-glycosidase F (PNGase F). The Western blot results showed that the HAs of untreated HA_P7_, HA_P6_, HA_P5_, HA_P4_, and HA_P3_ exhibited progressively greater mobility due to the lack of 1 NGS, respectively. However, the protein migration rate was same in all five HA mutants treated with PNGase F (Figure 3). This implies that variations in glycosylation level account for the band shifts in the untreated HAs and that these four putative NGSs at positions 210, 289, 304, and 21 are glycosylated.

### The effect of HA glycosylation patterns on the receptor affinity of h9.4.2.5-branched H9N2 AIVs

**Figure 4.**
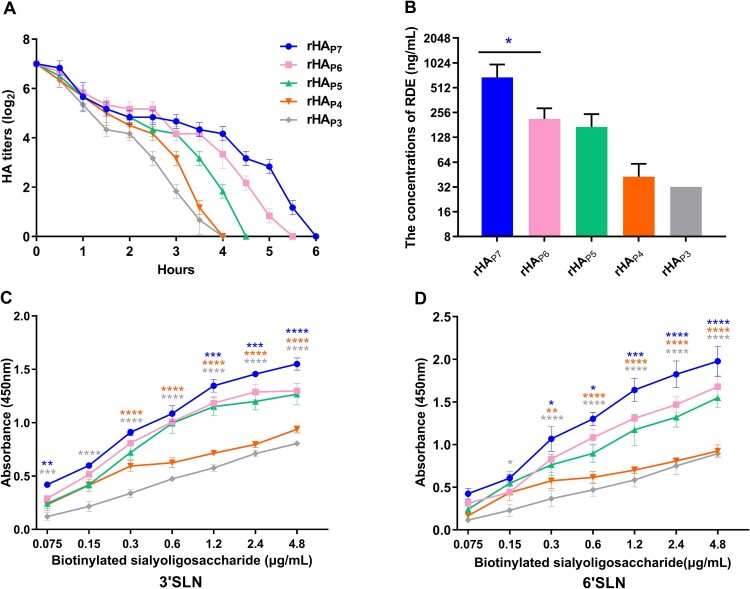
The receptor affinity of five virus mutants with different HA glycosylation patterns. (A) Twofold dilutions of 128 HAU of each virus were incubated at 4°C for 1 h with an equal volume of 0.5% chicken RBC. The samples were then transferred to 33°C, and the hemagglutination titres were measured at different time points. (B) 1% chicken RBCs were treated with twofold dilutions of RDE for 1 h, followed by an ice bath with an equal volume of 4 HAU virus for 45 min. The highest dilution of RDE that caused complete agglutination of RBCs was recorded. Each virus was incubated with 3′SLN (C) or 6′SLN (D) receptors to determine the receptor preference of HA. The significance of difference was analyzed by a Two-Way ANOVA test in GraphPad Prism 8.0.1 software. Each data point represents the mean ± SD of three independent experiments.

To clarify how HA glycosylation pattern variations affect viral receptor affinity, we rescued five virus mutants with distinct HA glycosylation patterns and named rHA_P7_, rHA_P6_, rHA_P5_, rHA_P4_, and rHA_P3_. The elution time of each virus from RBCs was then measured to assess the binding capacity of HA to chicken red blood cells (RBCs). The hemagglutination activity of these five virus mutants appeared to decline at 33°C after 30 min, as illustrated in Figure 4A. At 6 , 5.5 , 4.5 , 4 , and 4 h, respectively, they were fully detached from RBCs. This indicates that rHA_P7_ had the strongest capacity to bind to RBCs, followed by rHA_P6_, rHA_P5_, rHA_P4_, and rHA_P3_. The receptor-destroying enzyme (RDE) can remove SA receptors on RBCs. The viral receptor affinity can be indicated by determining the highest concentration of RDE that allows complete hemagglutination of RBCs. That is, the higher the concentration of RDE that allows complete hemagglutination, the better the receptor affinity. As shown in Figure 4B, rHA_P7_ can completely agglutinate RBCs treated with the highest concentration of RDE, followed by rHA_P6_, rHA_P5_, rHA_P4_, and rHA_P3_. In addition, we analyzed the receptor-binding preference of each mutant for avian-type (3′SLN) and human-type (6′SLN) receptors. These five virus mutants were able to bind to both 3′SLN and 6′SLN, as shown in Figure 4C and E, although rHA_P7_ and rHA_P6_ preferred 6′SLN (Figure S2).

### The effect of HA glycosylation patterns on the antigenicity of h9.4.2.5-branched H9N2 AIVs

**Figure 5.**
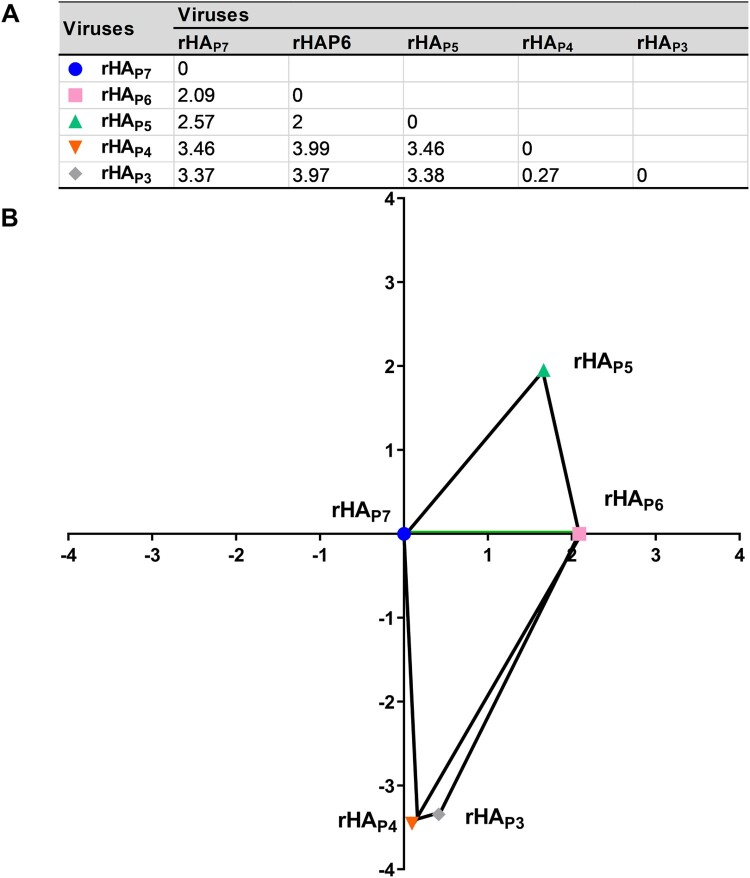
The antigenic map of five virus mutants with different HA glycosylation patterns. The 3 × 3 distance submatrix was extracted from the 5 × 5 distance matrix. The distance equation was then solved to compare the distances between any three viruses to obtain the x, y coordinates for the antigenic map.

To determine the impact of HA glycosylation patterns on viral antigenicity, we performed a cross-hemagglutination inhibition (HI) assay using chicken sera against each of the five virus mutants with distinct HA glycosylation patterns. As shown in Table S2, almost each serum showed the highest HI titre with its own virus mutant, while the cross-reactivity with others resulted in a 2log_2 _± 1log_2_ drop in HI titres. To more clearly visualize the antigenic distances between the five virus mutants in the two-dimensional plane, the antigenic distances between them were calculated based on the HI titres (Figure 5A), and the related antigenic map was presented (Figure 5B). The result showed a sequential rise in the antigenic distance between rHA_P6_, rHA_P5_, rHA_P3_, and rHA_P4_ and rHA_P7_.

### The effect of HA glycosylation patterns on the acid and thermal stability of h9.4.2.5-branched H9N2 AIVs

**Figure 6.**
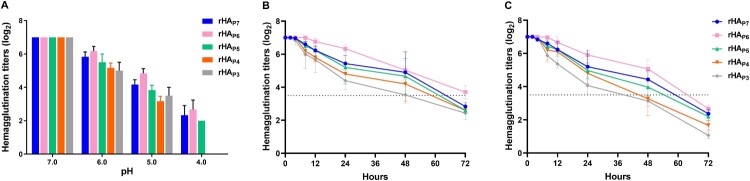
The stability of five virus mutants with different HA glycosylation patterns. (A) pH stability. 128 HAU/50 µL of virus was mixed with equal volumes of buffers (pH 4.0, 5.0, 6.0, and 7.0, respectively) and incubated at 37°C for 10 min. The sample was then assayed for hemagglutination titres. (B and C) Thermostability at 33°C and 37°C. 128 HAU/50 µL of virus was incubated at 33°C or 37°C for different time and then cooled to 4°C to determine the corresponding hemagglutination titres. Each data point represents the mean ± SD of three independent experiments.

The importance of HA glycosylation on acid stability and thermostability has been shown in previous studies [35, 36]. Acid stability is the basis for host adaptation of IAVs [4, 37]. Here, each of the five virus mutants was co-incubated at 37°C with equal volumes of different pH buffer, and their acid stability was evaluated by comparing hemagglutination titres. As Figure 6A illustrates, rHA_P7_, rHA_P6_, and rHA_P5_ retained their hemagglutination activity (HA ≥ 2log_2_) at pH 4.0, whereas rHA_P3_ and rHA_P4_ lost it, despite the fact that the hemagglutination titres of the five mutants dropped in acidic buffers in comparison to neutral buffers. In contrast, the highest acid stability was demonstrated by rHA_P6_, followed by rHA_P7_ and rHA_P5_, while rHA_P3_ and rHA_P4_ were among the lowest.

Temperature, an inherent environmental and host parameter, has a considerable influence on IAV invasion and host tropism [38, 39]. To assess the thermostability of the five virus mutants, each was subjected to a 33°C or 37°C treatment and sampled at specific time points for a hemagglutination assay. As illustrated in Figure 6B and C, the hemagglutination activity of each mutant steadily reduced with longer treatment times at the same temperature or with higher treatment temperatures over the same time period. After 72 hours of incubation at 33°C (Figure 6B), the hemagglutination titre of rHAP6 maintained more than 50% of the initial titre, but after 48–72 hours, the titres of the other strains fell to less than 50% of the original titre. Except for rHAP4 and rHAP3, whose haemagglutination titres fell below 50% of the original titre after 48 hours of incubation, the titres of the remaining strains began to fall below 50% of the original titre after 60–72 hours of incubation at 37°C (Figure 6C). As a result, it can be concluded that rHA_P6_ had the best thermostability, followed by rHA_P7_, rHA_P5_, rHA_P4_, and rHA_P3_.

### The effect of HA glycosylation patterns on the multiplication of h9.4.2.5-branched H9N2 AIVs

**Figure 7.**
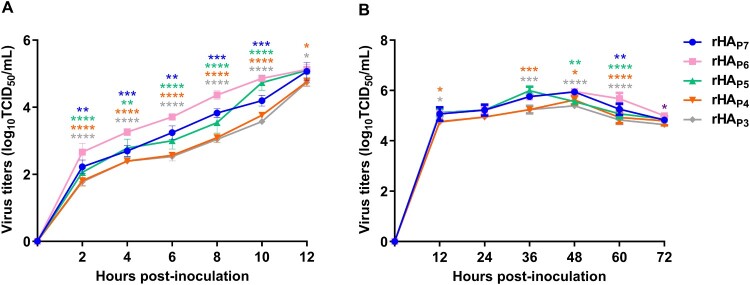
The growth kinetics of five virus mutants with different HA glycosylation patterns. CEF cells were infected with each virus at an MOI of 0.1 or 0.01, and cell supernatants were collected at different time points to determine the TCID_50_. One-step (A) and multi-step (B) viral growth kinetic curves were plotted. The significance of difference was analyzed by a Two-Way ANOVA test in GraphPad Prism 8.0.1 software. Each data point represents the mean ± SD of three independent experiments.

The capacity to multiply is a crucial phenotypic indicator of IAV fitness [8]. To investigate the effects of HA glycosylation patterns on viral multiplication in vitro, CEF cells were infected with each of the five virus mutants at an MOI of 0.1 or 0.01 to plot growth curves. The one-step growth kinetic curves showed that the titre of rHA_P6_ was significantly higher than that of the other four virus mutants at 2–10 h post-inoculation (hpi) (*p* < 0.01–0.0001) and higher than rHA_P4_ and rHA_P3_ at 12 hpi (*p* < 0.05) (Figure 7A). According to the multi-step growth kinetic curves (Figure 7B), the five virus mutants were able to reach peak titres between 36 and 48 hpi. The titre of rHA_P6_ was also noticeably greater at 60 hpi than that of the other four virus mutants (*p* < 0.01–0.0001).

## Discussion

H9N2 AIVs have evolved in birds and poultry worldwide for more than half a century [16, 17], highlighting the necessity to pay close attention to their evolutionary fitness. Since 2007, h9.4.2.5-branched AIVs have dominated the H9N2 AIV population (Figure 1 and Table 1). Despite numerous efforts, this branch of viruses still continued to be prevalent in immunized flocks [21, 27, 40]. What enables them to have such powerful fitness is a question worth exploring. The HA glycosylation modifications play a critical role in viral adaptive evolution [6, 10, 11]. For h9.4.2.5-branched AIVs, their HA glycosylation has evolved over time. The first glycosylation pattern, P7, with seven NGSs, appeared in 2007. Thereafter, the number of NGSs on HA gradually decreased, with the four major patterns P6, P5, P4, and P3 appearing one after the other. Interestingly, the epidemiological dominance of different patterns of viruses has drifted over time. For instance, those viruses with the P7 pattern had a clear epidemiological advantage until 2012. This dominance was overthrown, though, by viruses with the P6 pattern, and they have maintained it ever since. The other pattern viruses, on the other hand, have essentially vanished after 2018 (Figure 2). These findings prompted us to investigate the effects of HA glycosylation patterns on the adaptive evolutionary phenotypes of h9.4.2.5-branched H9N2 viruses, such as viral receptor affinity, antigenicity, stability, and multiplication.

The binding of HA to SA receptors plays a well-defined role in mediating the adsorption and endocytosis of IAVs [3]. There are up to seven NGSs on HAs of h9.4.2.5-branched viruses, two of which are located at positions 128 and 210 in the HA header, with the remaining five located at positions 21, 289, 296, 304, and 483 in the HA stem (Figure S3). The addition of N-glycans in the HA globular domain often results in a decrease in receptor avidity [12]. However, our results showed that rHA_P7_ with seven NGSs exhibited a greater receptor affinity in the five virus mutants with different HA glycosylation patterns (Figure 4). The first HA glycosylation pattern to emerge in the h9.4.2.5-branched viruses was seven NGSs, which might be beneficial for enhancing viral receptor affinity and immune escape [41, 42]. However, the phenotypic advantage of viruses with this glycosylation pattern was overtaken by those viruses with six NGSs as soon as 6 years later (Figure 2), a striking manifestation of which was the greater ability of rHA_P6_ to multiply in CEF cells than rHA_P7_ (Figure 7). In contrast to rHA_P7_, rHA_P6_ removes the NGS located at position 210 on the HA head (Figure 2), which is close to the receptor-binding and antigenic sites (Figure S3). Although elimination of the glycan at position 210 reduces the receptor affinity of HA, it appears to be especially crucial to increase the fitness of h9.4.2.5-branched AIVs. An excessively high affinity for HA can cause IAV to bind to cellular receptors too firmly, which might be detrimental to the viral fitness. In this case, it requires a neuraminidase (NA) with stronger enzymatic activity to match this HA to facilitate the movements of infectious virions on the cell surface to reach the ideal sites. Furthermore, the release of progeny virions also requires a functional balance between HA and NA [43–45]. Therefore, it is necessary to compare the activity of NAs matching the HAs with five glycosylation patterns in order to further assess the effects of HA/NA functional balance on the fitness of h9.4.2.5-branched AIVs. Human infections with H9N2 AIVs have been reported sporadically since 1998. Given that the rHA_P7_ and rHA_P6_ preferred 6′SLN over 3′SLN (Figure 4C-D and Figure S2), indicating their potential for cross-species transmission to humans, we further analyzed the HA glycosylation patterns of 72 human-derived H9N2 viruses isolated in China from 1998 to 2024. The results showed that 58 of the 72 human isolates belonged to the h9.4.2.5 branch (Figure S4), and 54 human isolates possessed the HA glycosylation pattern P6 (Table S3). This indirectly reflects the evolutionary advantage of h9.4.2.5-branched AIVs and the dominant potential with six NGSs.

IAVs are able to evolve rapidly to escape pre-existing immunity in a process known as “antigenic drift” to maintain their fitness [8]. Due to the economic damage caused by enzootic H9N2, many countries have introduced vaccination of poultry at either a national or local level to try to control H9N2 infection. Unlike human influenza vaccines, however, H9N2 vaccines are generally not regularly evaluated for their efficacy against antigenically drifted viruses and are hence updated less often. Limited evidence showed that antisera to H9N2 vaccines are very effective against earlier strains but not more recent variants [46], which leads to the spread of the latter among the vaccinated chickens [46, 47]. While HA glycosylation has been extensively studied in defining IAV antigenicity [48–52], less emphasis has been paid to how differences in HA glycosylation patterns affect the antigenicity of vaccine strains. Here, we found that only six of the fifteen H9N2 vaccine strains that were utilized in China shared the same HA glycosylation patterns as P7 or P6 of h9.4.2.5-branched AIVs, respectively (Table S4). In addition, the antigenic distances between the five virus mutants, rHA_P7_ to rHA_P6_, rHA_P5_, rHA_P3_, and rHA_P4_, were displayed in increasing order according to the antigenic profiles (Figure 5). This means that alterations in the glycosylation pattern of a given strain may lead to antigenic drift, enabling both the parental and mutant strains to escape immunity to one another. Consistent with the findings of Boyoglu-Barnum et al [53], our study also suggests that glycosylation of the HA stem contributes to viral antigenicity, despite the fact that glycosylation of the HA globular head is the primary driver of IAV antigenic drift [48, 54]. It should be emphasized that the degree of HA glycosylation and the type of glycans attached to HA are host – or cell-dependent [55]. IAV antigenicity can be affected by changes in glycosylation patterns when replicated in different hosts or cells. It is therefore recommended to use either chicken embryos or the same cells for vaccine strains.

IAVs enter cells by HA-mediated membrane fusion. The conformational state of HA switches from a native structure to a fusion-active conformation when exposed to the acidic environment of the cellular endosome, which thus initiates membrane fusion. The acidic and thermal stability of HA plays a crucial role in the ability of IAVs to adapt to complex and changing external and intra-host settings [56], which determines the host range, infectivity, transmissibility, and pandemic potential of IAVs [4, 37–39]. There is variation in the pH threshold between strains and subtypes of IAVs that triggers the fusion of HA. In general, the average pH value of HA activation in poultry AIVs is lower than that of wild bird AIVs [4]. Lowly pathogenicity H9N2 AIVs have a lower HA fusion pH (5.3–5.5) than highly pathogenic H5N1 and H7N9 strains (5.6–6.0) [4, 37]. The activation pH values of the H1 subtype of swine influenza viruses are lower than those of the H3 subtype [4]. Human pandemic H1N1 influenza viruses that emerged in 2009 had a fusion pH of 5.5, whereas human seasonal H1N1 influenza viruses those recovered from 2010–2012 ranged from 5.2–5.4 [4]. An HA protein with a lower activation pH is more stable in the cellular endosome and more resistant to extracellular inactivation [57–59]. Low pH is the biological trigger for HA activation, however, other destabilizing factors, such heat, can also cause HA refolding [4]. In this study, rHA_P6_ with six NGSs showed the best acid and thermal stability, followed by rHA_P7_, rHA_P5_, rHA_P4_, and rHA_P3_ (Figure 6). This is a further potential explanation for how, in just three years following their initial appearance in 2010, the number of strains with six NGSs at positions 21, 128, 289, 296, 304, and 483 on HA was able to surpass that of strains with seven NGSs, and how they been dominating the h9.4.2.5-branded H9N2 AIVs ever since. It offers more proof that HA glycosylation patterns affect viral dominance in epidemiology. Furthermore, our study suggests that when HA glycosylation accumulates to a certain extent, non-conserved glycosylation sites may mutate into non-glycosylation sites to maintain the functional homeostasis of HA and thus viral fitness, which is the driving force behind the changes in the number of NGSs on HA.

All of our results together show that the number of NGSs on HA experienced a dynamic adjustment during the evolution of h9.4.2.5-branched H9N2 AIVs, which influences viral stability and multiplication in addition to regulating viral receptor affinity and antigenicity. This effect is especially evident in the dominance drift between H9N2 viruses with seven and six NGSs on HA. Therefore, viruses with different HA glycosylation patterns either adapt, evolve, or become extinct when faced with new selective pressures. Although we investigated the effects of the dynamic changes in HA glycosylation patterns on the fitness of h9.4.2.5-branched H9N2 AIVs, there are still issues that need to be addressed when interpreting the findings, including (i) the type and structure of site-specific glycan, (ii) the “compensatory effects” of epistatic mutations at other HA sites, (iii) the HA/NA functional balance, and (iv) the contribution of other internal genes. This would be a boon to efforts to elucidate the relationship between HA glycosylation and the adaptive evolution of h9.4.2.5-branched H9N2 AIVs and to predict the potential for viral immune evasion. It would also facilitate the development of novel influenza vaccines that take advantage of glycosylation-dependent mechanisms.

## Data Availability

All study data are included in the article and/or supplementary material.
